# MicroRNAs as Regulators of Phagocytosis

**DOI:** 10.3390/cells11091380

**Published:** 2022-04-19

**Authors:** Wojciech Gierlikowski, Barbara Gierlikowska

**Affiliations:** 1 Department of Internal Medicine and Endocrinology, Medical University of Warsaw, Banacha 1a, 02-097 Warsaw, Poland; 2 Department of Laboratory Diagnostics and Clinical Immunology of Developmental Age, Medical University of Warsaw, Żwirki i Wigury 63a, 02-091 Warsaw, Poland; barbara.gierlikowska@wum.edu.pl

**Keywords:** microRNA, phagocytosis, macrophage, microglia, cytokine, phagosome, phagolysosome, reactive oxygen species, retinal pigment epithelium, osteoclast

## Abstract

MicroRNAs (miRNAs) are short non-coding RNAs that regulate gene expression and thus act as important regulators of cellular phenotype and function. As their expression may be dysregulated in numerous diseases, they are of interest as biomarkers. What is more, attempts of modulation of some microRNAs for therapeutic reasons have been undertaken. In this review, we discuss the current knowledge regarding the influence of microRNAs on phagocytosis, which may be exerted on different levels, such as through macrophages polarization, phagosome maturation, reactive oxygen species production and cytokines synthesis. This phenomenon plays an important role in numerous pathological conditions.

## 1. MicroRNAs—Biogenesis, Genomics, Regulation, Mechanisms of Action and Biological Functions

MicroRNAs (miRNAs) are single-stranded RNAs of about 22 nucleotides that take part in post transcriptional regulation of gene expression. MicroRNAs were discovered in the 1990s when a regulation role of *lin-4* gene in the development of Caenorhabditis elegans was described. The authors showed that *lin-4* does not encode for protein but is transcribed into a 22nt product that targets a complimentary sequence in the 3′UnTranslated Region (3′UTR) of *lin-14* mRNA. Basing on antisense RNA-RNA interaction, this phenomenon leads to downregulation of the Lin-14 protein resulting in proper larval development [1,2]. In the following years it was shown that similar small RNAs are present and conserved among other species [3], including more than 2500 identified human microRNAs, as deposited in the miRbase database (v22) [4]; importantly, most of those microRNAs are conserved throughout mammals [5]. This led to the conclusion that such a mechanism of regulation of gene expression is common throughout different organisms and not limited to genes involved in development. Subsequently, the term microRNA was introduced [6]. In animals, binding of a microRNA with a 3′UTR is based on 6–8 nucleotides at the 5’ end of the microRNA, called the seed region. Such an interaction typically leads to translation repression without mRNA cleavage [7]. However, it was demonstrated that overall mRNA destabilization accounts for most microRNA-mediated repression, which makes presenting changes at the mRNA level sufficient for proving a significant influence of a given microRNA on its predicted target [8]. Most human genes are targeted by microRNAs [5]. The effect of a single microRNA on protein expression is rather modest, leading to fine-tuning of production [9]; however, a single microRNA can target numerous mRNAs, and a single mRNA can be targeted by numerous microRNAs [10]. What is more, a single microRNA may target different genes involved in a single biological process [11], potentially leading to significant changes in cell function.

Genes encoding for microRNAs are spread throughout the genome [12] and are transcribed mainly by RNA Polymerase II (Pol II) as pri-miRNAs [13]. Each pri-miRNA forms at least one hairpin structure, which is modified by a microprocessor complex formed by DROSHA and two DiGeorge Syndrome Critical Region 8 (DGCR8) proteins [14], resulting in about 60nt stem-loop structure called a pre-miRNA [15] that is subsequently exported from the nucleus to the cytoplasm by Exportin 5 and Ran [16]. Further processing is performed by Dicer [17], and results in cutting off the loop, which creates a miRNA duplex [18]. The duplex is subsequently loaded into Argonaute proteins, forming an RNA-induced silencing complex (RISC) [19]. Each strand of the duplex is a mature microRNA, and each RISC uses a single strand as a guide strand, although with preference for one of the microRNAs [20]. Nevertheless, both products from the 5′ and 3′ end of the stem-loop structure can be used as guides to further regulate gene expression [21]. Upon forming the RISC, the microRNA can exert its functions for days [22].

**Figure 1:**
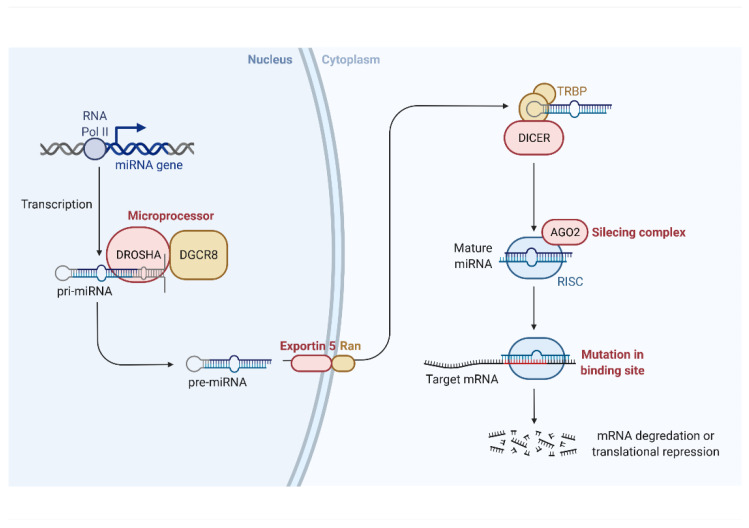
Schema of biogenesis of microRNA. MicroRNA-encoding genes are transcribed by RNA Polymerase II as pri-miRNA, which contain a hairpin structure. Pri-miRNA are further modified by the microprocessor complex, consisting of DROSHA and two DiGeorge Syndrome Critical Region 8 proteins, to form pre-miRNA. After transfer to the cytoplasm, mediated by Exportin 5 and Ran, the loop is cut off by DICER. The MiRNA duplex is than loaded into Argonaute proteins (AGO), forming an RNA-induced silencing complex (RISC). One of the miRNA strands is degraded in a single RISC, but each of them can be used as a guide to identify target mRNA, leading to transcription repression or mRNA degradation.

MicroRNA biogenesis, genomics, regulation, mechanisms of action, target recognition, and biological functions were reviewed by Bartel [23]. General information is summarized in Figure 1.

Experimental modulation of microRNAs is relatively easy. Overexpression can be obtained by microRNA-mimicking particles, available commercially, or by using vector systems. A few methods of silencing selected microRNAs have been introduced, such as 2’-O-methyl oligonucleotides [24], locked nucleic acids [25], and antagomiRs [26], which are all generally chemically engineered oligonucleotides, or microRNA sponges, which are expressed from transgenes in transfected cells [27], or viral vectors [28].

MicroRNAs were quickly shown to be deregulated in numerous diseases. Much of the attention has been focused on aberrant microRNA expression in cancer, including studies on employing microRNAs in diagnostics, monitoring and treatment [29]. Usefulness of microRNAs as biomarkers is of special interest, as they may be measured in different body fluids, including blood [30], thus being useful in non-invasive tests [31].

It was postulated that microRNAs may be present in blood within exosomes, a type of extracellular vesicle, and that microRNAs can be transferred between cells [32], thus acting in paracrine manner [33] (as reviewed in [34]). This phenomenon was observed also for immune cells ([35,36,37]; reviewed in [38]). Numerous exosomes are needed for transport of a biologically significant number of microRNA particles [39].

Differentiation of hematopoietic progenitor cells in bone marrow depends on numerous transcription factors (as reviewed in [40]). Proper differentiation is based on continuous interplay between transcription factors and microRNAs. MicroRNAs may be regulated by transcription factors upon stimulation of immune cells (revised in [41]). Consequently, we postulate that a minimal set of data when investigating regulation of a given target by a chosen microRNA should involve (1) microRNA deregulation in certain situations, (2) in silico predicted binding of the microRNA with 3′UTR of the target’s mRNA, and (3) appropriate change in the target’s expression, at least at the mRNA level. Assessing the influence of microRNAs on their target may be more difficult in the setting of the antiviral innate immune response, as it was proven for macrophages that this leads to alternative polyadenylation resulting in 3′UTR shortening and loss of microRNA binding sites [42]. In this review we include studies that investigate the effect of certain microRNAs on phagocytosis, even without specifying a target gene. The lacking information may serve as a direction for further research.

Therapies based on nucleic acids were recently introduced in clinical use. They can be divided into DNA-based and RNA-based therapies. DNA-based therapies raise safety concerns due to potential integration into the host genome, despite some studies demonstrating that in the case of intramuscular administration the risk is negligible [43]. RNA-based therapeutics do not even enter the nucleus. Their use may be based on RNA interference [44] or mRNA [45]. Despite some obstacles, such as degradation of RNA by RNases, difficulties in delivery across the cell membrane, or immunogenicity of exogenous RNA that causes cell toxicity and impaired translation into therapeutic proteins, some RNA-based drugs have been developed and are currently available on the market. Additionally, some of the drugs currently being developed are targeted to stimulate immune responses toward solid tumors, as reviewed by Damase [46]. Miravirsen, a locked nucleic acid-modified antisense oligonucleotide targeting miR-122, was the first microRNA-based therapeutic that entered a phase 2 clinical trial. The history of development of this anti-HCV drug underlies several additional difficulties in the discovery of such compounds [47]. Trials to “supplement” microRNAs to restore their function and modulate intercellular pathways, as in the case of miR-34a, targeting numerous oncogenes, are also in progress [48]. Despite high putative potential, no microRNA-based therapy has been introduced into clinical practice. Nevertheless, some other RNA-based therapies have been approved by medical agencies (as reviewed in [49]).

## 2. The Immune System and MicroRNAs

The immune system is an advanced network of biological processes. They are functionally related to each other, which increases the chance of appropriate response to pathogen, or other stimuli. The innate immune system provides a first-line, non-specific response to a wide spectrum of stimuli, whereas the adaptive immune system ensures a precise response to stimuli by learning to recognize molecules it has previously encountered [50].

The regulation of innate immune cells by microRNAs, including myeloid cell development and its functional modulation, has been previously discussed [51,52]. Mehta et al. expanded the study, assigning the microRNAs to selected innate immune cells. In this context, the authors focused on three cell types: macrophages, granulocytes, and natural killer cells. Both macrophages and granulocytes are called professional phagocytes, and each may be a cellular target for microRNAs, but only macrophages are considered as appropriate for potential experimental transfection [53]. The promising usefulness of macrophages inspired us towards a detailed analysis of the discoveries made using this cellular model.

Macrophage development is regulated by miR-155 and miR-146a expression [54,55,56,57]. miR-155 is strongly induced by NF-κB and activator protein 1 (AP-1) in response to cytokine appearance and TLRs activation [55]. LPS stimulation also activates AKT1, causing repression of miR-155 and miR-125b, and induction of let-7e, which negatively regulates TLR4. Interestingly, miR-155 is also repressed by IL-10 signaling through STAT3 [57]. miR-146a is also induced by NF-κB and negatively regulates TLR signaling by targeting TRAF6 and IRAK1 [58]. A working hypothesis is that these microRNAs may act antagonistically to produce a dynamic and coordinated inflammatory response. In addition to its function in immunity, miR-155 drives cell differentiation [59]. 

Expression of microRNAs in macrophages can be dynamically regulated in response to different stimuli, such as antigen recognition by cell receptors, NF-κB activation and cytokine release [55,58,60]. Thus, pathogen infection, phagocytosis or developing inflammation are directly dependent on it. In our work we, for the first time, precisely summarize microRNAs involved in phagocytosis and phagocytosis-related processes.

The adaptive immune system mostly comprises B cells and T cells, which together provide a targeted second line of immune defense against foreign pathogens after priming from the innate immune system. Comprehensive reviews of all the microRNAs implicated in adaptive immunity can be found elsewhere [60,61,62,63,64,65]. Here, we focus on the most recent discoveries and emphasize key examples that highlight either unique facets of microRNA biology or network motifs by which microRNAs can influence phagocytosis—the most important mechanism of the innate response.

## 3. Phagocytosis—Overview

The phenomenon of phagocytosis was first described by zoologist Ilya Mechnikov. He made his groundbreaking observations in Messina, Sicily. One day, Mechnikov observed starfish larvae under a microscope. In one of these larvae he saw a splinter surrounded by cells that were not identified at the time. Thoughts flashed through his mind that the body wanted to get rid of the intruder and that perhaps it could protect itself in a similar way against other pathogens. To confirm that this observation was not accidental, he tore off a few rose thorns and stuck them into the larvae of starfish found on the beach. The next day, he observed under a microscope that the spines were recognized by cells trying to remove them. Mechnikov did not have a medical education, but by chance he received a dissertation by another prominent researcher, Cohnheim. He described inflammation as a defense reaction in which the vessels dilate and leak cells from them. This time, Mechnikov was sure of his theory—inflammation serves to attract cells into the tissues, which were soon called phagocytes [66,67]. While lower organisms use phagocytosis for the absorption of nutrients, phagocytosis in higher organisms occurs mainly in specialized phagocytic cells such as macrophages and neutrophils, and it has evolved into an extraordinarily complex process underlying an important biological mechanism mainly of the immune response [68].

Although molecules and cells of various origins can be phagocytosed, the phagocytosis of microorganisms is the most important in human immunity. Phagocytosis is initiated via the recognition and binding by appropriate receptors of the phagocytic cell with the molecule or cell which is to undergo phagocytosis. This process of facilitating phagocytosis is called opsonization, and factors facilitating and enhancing phagocytosis are referred to as opsonins. The most important opsonins are immunoglobulins and complement components [69] coating the particle antigen, or receptors that bind directly to surface determinants of pathogens, such as mannose receptors, scavenger receptors or dectin-1 [70]. Subsequent receptor clustering causes a signaling cascade that results in a transient burst of actin polymerization, forming the phagocytic cup from which pseudopods extend to engulf particle. Actin polymerization continues at pseudopod tips, while depolymerization of the actin at the base of the phagocytic cup occurs when pseudopod tips meet, allowing for closure the cup [71,72]. This initiates a signal transduction cascade that ultimately results in membrane reconstruction, a process directed by massive cytoskeleton rearrangements accompanied by active focal endocytosis [71]. Sequential fusion events with components of the endocytic pathway, together with fission of vesicles and tubules, remodel the phagosome and initiate its degradative properties while sorting out cargo and membrane components for cellular recycling. This leads to fusion of the lysosome and phagosome creating a phagolysosome [73], a degrading organelle with strong microbicidal activity [74]. The newly formed phagolysosomes are characterized by high activity of endosomal and lysosomal hydrolases [75] that enhance the effective killing of pathogens [76,77]. The killing of pathogens in phagolysosomes also depends on the activation of NADPH oxidase (Nox2). Nox2 catalyzes the formation of a highly unstable superoxide anion (O_2_^−^), which initiates a variety of chemical reactions leading to the formation of reactive oxygen species (ROS) such as peroxides, hydroxyl radicals, and singlet oxygen [78]. ROS, in addition to their important role in killing pathogens, may lead to damaged proteins, lipids and DNA [79,80]. At the end of the phagocytosis process, the non-digested material is expelled, assimilated, or some antigens may be presented to other immune cells [81].

Phagocytosis is an extremely effective process, but in the literature has been documented as a limiting factor. According to Jubrail et al., the number of pathogen particles per single cell (MOI) is of key importance. At low values of this ratio, as observed in the early phase of infection, immune cells can kill almost all absorbed pathogens, but the ability to phagocytose decreases with increasing bacterial load [82].

To sum up, phagocytosis refers to the receptor-mediated uptake of large particulate matter. Particle engulfment is notably efficient in specialized myeloid cells, namely macrophages and neutrophils [73]. These cells constitute the first line of defense against invading microorganisms [83]. However, it should also be mentioned that phagocytes are capable of linking innate and adoptive immune systems. By presenting antigens derived from phagocytic pathogens, phagocytes activate lymphocytes [81]. Less appreciated is the essential role of phagocytosis in the maintenance of tissue homeostasis. Professional and non-professional phagocytes remove billions of apoptotic cells daily [84]. This process, known as efferocytosis, plays an important role in wound healing [85].

### Examples of Cells with Phagocytic Capacity

**Figure 2:**
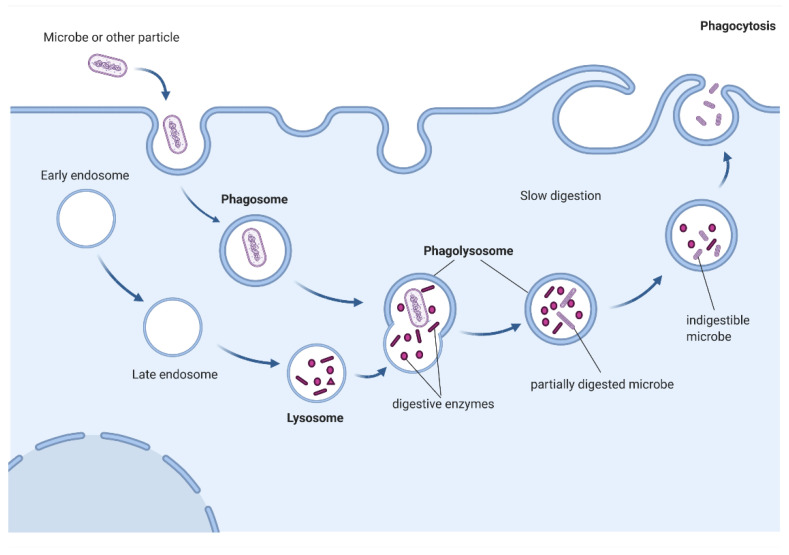
Schema of phagocytosis performed by macrophages. Macrophages are professional phagocytes and are highly specialized in removal of pathogens, dying or dead cells and cellular debris. They differentiate from hematopoietic stem cell and polarize, depending on the cytokine profile, into different types, among which mainly M1 macrophages are involved in phagocytosis of bacteria (not shown). The removal of bacteria is initiated by activation of specified receptors that facilitate capture and uptake of particles. When a macrophage ingests a pathogen, the pathogen becomes trapped in a phagosome, which then fuses with a lysosome formed by endosomes. Within the phagolysosome, enzymes and toxic peroxides digest the pathogen. Waste material is expelled or assimilated. Pathogen epitopes can also be presented via major histocompatibility complex class II proteins, thus taking part in an adaptive immune response.

Although macrophages and neutrophils are classified as professional phagocytes, and use similar mechanisms for the internalization of targets, there are significant differences between their mechanisms of action. Nordenfelt at al. showed that the main differences concern biochemical and structural changes inside both types of cells. Both cell types perform FcγR-mediated pseudopod extension and the complement receptor-mediated mechanism, during which recognized targets are absorbed inside the phagocyte. However, macrophages are known to have a significantly wider spectrum of Pattern Recognition Receptors (PRRs) [86]. Moreover, the differences also involve the mechanism of phagosome maturation: (1) neutrophils require granule delivery for phagosome formation, but macrophages form phagosome through endosomal pathway; (2) Rab5 expression occurs in neutrophilic phagosomes (the protein characteristic for early-formed phagosomes), while phagosomes of macrophages express Rab5 and Rab7 protein (characteristic for late-formed/matured phagosomes); (3) in contrast to the neutral pH of neutrophilic phagosomes (pH 7), macrophages require acidification of the phagosome (pH 4–5) to increase enzyme activity [87]. Another difference between the types of phagocytes concern membrane trafficking and targeting of absorbed particles. Neutrophils involve a granule-dependent targeting pathway, while macrophages have Ca^2+^-independent lysosome targeting and fusion with lysosomes [86]. Phagocytosis performed by macrophages removes microbes, dead cells, and tissue debris; thus, macrophages become an essential component for maintenance of tissue homeostasis [88]. A schema of phagocytosis performed by macrophages is presented in Figure 2.

Phagocytic properties of macrophages are even more important, as those cells are widely distributed in the human body, presenting a variety of morphological and functional phenotypes [89]. Macrophages can be derived from progenitors in bone marrow and fetal precursors in the yolk sac. Macrophages that are resident in bone are termed osteoclasts [90], whereas those derived from the yolk sac include Langerhans cells (tissue-resident macrophages of the skin), Kupffer cells (in the liver), microglia (in the brain), alveolar macrophages (in the lung), red pulp macrophage (in the spleen) and others present in pancreas or kidney [89]. Macrophages are involved in remodeling and functionality of the above-mentioned tissues [91,92] and regulation of angiogenesis [93,94]. Remembering that macrophages are an important component of the immune response, their dual functionality must be mentioned. During injury and pathogen infection, macrophages polarize to the M1-like phenotype. M1-like phenotype macrophages have increased expression of distinct phagocytic receptors, such as FcγRI, FcγRII, and FcγRIII, allowing for the enhanced clearance of taken up particles [95,96,97,98]. Monocyte differentiation potential into the M1 or M2 phenotype is highly dependent on the surrounding microenvironment. Exposure to LPS or IFNγ induces the differentiation of M1-like macrophages, whereas addition of IL-4 induces the differentiation of M2-like macrophages [99]. These results suggest that monocytes are phenotypically polarized by the microenvironment to confer on them specific functions. In many inflammatory diseases, uncontrolled polarization to the M1-phenotype may lead to exacerbation of the disease [100].

Phagocytosis is indirectly modulated by NF-κB activation and furthers proinflammatory cytokine synthesis [101]. Cytokines modulate phagocytic functions of immune cells; thus, the appropriate cytokine concentration may enhance pathogen eradication. For example, low amounts of TNF-α and IL-1β increase phagocyte chemotaxis, enable intracellular phagosome maturation, and stimulate phagolysosome formation, necessary for final intracellular killing [102]. TNF-α, IL-1β, IL-6, IL-8 may activate oxidative and non-oxidative metabolic responses of immune cells to pathogens [103]. INFs and TGF-β have been identified as stimulators of phagolysosome formation [104]. The appropriate concentration of cytokines promotes a proper immune response, but overproduction or prolonged exposure of leukocytes to pro-inflammatory cytokines may exacerbate inflammation. 

Some cells (excluding macrophages and neutrophils) have intermediate phagocytic capacity, such as bladder epithelium or thyroid cells, both capable of phagocytosis of erythrocytes. Other examples of non-professional phagocytes are retinal epithelial cells, which internalize the distorted ends of retinal rods [68,105], and vascular smooth muscle cells, which take part in the development of atherosclerotic plaque [106]. The phagocytic capacity of the described cells is based on the presence of phagocytic receptors, dynamics of membrane trafficking, actin cytoskeleton rearrangements, and signal transduction [68]. All phagocytic cells play an important role in the immune response, increasing the effectiveness of the described process at various stages.

## 4. Role of MicroRNA in Regulation of Different Stages of Phagocytosis Performed by Macrophages

Phagocytosis performed by macrophages is one of the fundamental components of the innate immune response [68]. It can be divided into several steps, and microRNAs play a role in the regulation of all of them through regulation of numerous proteins, as shown in Table 1 and discussed below.

### 4.1. Differentiation

The initial process is differentiation of hematopoietic stem cells into monocytes/macrophages. Not surprisingly, microRNAs take part in this process [184]. It was demonstrated that miR-22, whose expression is upregulated by PU.1 transcription factor, targets MECOM (EVI1), which further increases c-Jun but decreases GATA2 expression. Downregulation of miR-22 may appear in acute myeloid leukemia and block differentiation of bone marrow blasts; restoration of its expression relieves the block. Expression of miR-22 in HL60 and THP1 cell lines increases upon phorbol myristate acetate (PMA) treatment [185]. PU.1 represses the miR-17-92 cluster promoter, which encodes for miR-17, miR-18a, miR-19a, miR-20a, miR-19b-1, and miR-92a, through histone demethylation [186]. PU.1 upregulates miR-424, which suppresses the transcriptional factor NFI-A, subsequently stimulating monocyte differentiation through differentiation-specific genes such as M-CSFr [187].

### 4.2. Polarization

Polarization to M1 macrophages is related to their inflammatory functions. Thus it is necessary for performing phagocytosis. Transition from the M1 to M2 phenotype is essential to resolve inflammation [188]. Both phenotypes play important roles in phagocytosis, and both are promoted by different stimuli such as bacterial components, cytokines, or other molecules.

The M1 phenotype is stimulated by lipopolysaccharide (LPS) and proinflammatory cytokines, such as INF-γ and TNF-α. Expression of the M1 phenotype is regulated by transcription factors such as NF-κB, IRF-3, and IRF-5 [189,190,191]. In turn, the appearance of anti-inflammatory cytokines, such as IL-10, IL-4 or IL-13, promotes the M2 phenotype, which is coordinated by the STAT-6 transcription factor [192,193,194]. The phenotypes may also be stimulated by up or downregulation of microRNAs. Most documented microRNAs are involved in the promotion of the pro-inflammatory phenotype—M1. The specify of polarization is presented and the most important studies are discussed below.

Graff et al. used substances to differentiate macrophages into M1, M2a, M2b, and M2c (by IFNγ and LPS, IL-4, IgG and LPS, TGF-β1, respectively) and employed microarrays to check changes in microRNAs profiles (with subsequent verification using TaqMan assays). They noticed increases of miR-125a-3p, miR-155-5p, miR-155-3p in M1, miR-193b in M2a, miR-27a-5p, miR-155-5p, miR-155-3p in M2b and decreases of miR-26a-2-3p, and miR-29b-1-5p in both M1 and M2a. Interestingly, upon LPS stimulation, miR-27a-3p and miR-222-5p were decreased in M1 but increased in M2b [195], which illustrates that a balance between multiple microRNAs may be an additional important factor.

Cobos Jiménez et al. employed next-generation sequencing (NGS) to compare microRNA expression in monocytes and differentiated macrophages. Expression of miR-34a-5p, miR-106-3p, miR-132-3p, miR-335-5p, miR-362-3p, and miR-424-5p was upregulated in macrophages compared with monocytes. MiR-145-5p was uniquely upregulated in M1, whereas miR-181b-5p was uniquely downregulated in M2a cells, and miR-200a-3p was exclusively downregulated in M2c macrophages. MiR-146a-5p, miR-193a-5p, and miR-29b-3p were upregulated, and miR-629-5p was downregulated in M1 cells. miR-500a-5p and miR-502–3p were upregulated in M2a macrophages; miR-21-5p, miR-22-3p, and miR146b-5p were upregulated, and miR-339-3p was downregulated in M2c cells. MiR-221-3p, miR-222-3p, and miR-511 were highly expressed in M2a macrophages. M1 macrophages present high expression of miR-146a-5p, miR-29b-3p, and miR-147b, and low expression of miR-221-3p. M2a macrophages display high levels of miR-193b-3p and miR-511, and low expression of miR-181a-5p and miR-181b-5p. M2c macrophages express high levels of miR-125b-5p, miR-125a-5p, and miR-99b and uniquely low levels of miR-200a-3p [196].

Contrary to the abovementioned NGS results, Das et al. reported that upon *Leishmania donovani* infection, miR-146a-5p is upregulated in a BET bromodomain protein 4 (BRD4)/p300-depdendent manner (in this conditions miR-181a-5p and miR-125a-5p are upregulated too, whereas miR-26a-5p is downregulated, which promotes M2 over M1 polarization). They showed that silencing of miR-146a-5p resulted in downregulation of M2 markers (YM1, FIZZ1, CCR7, Arg1, pSTAT6 and c/EBPβ) and upregulation of p-STAT1, IRF-1, AP1, TRAF6, IRAK1, which resulted in an increase in IL-12 and TNFα production [153]. Huang et al. also reported that miR-146a promotes polarization towards the M2 phenotype [152]. Similar observations were carried out by Peng et al. The authors confirmed that miR-146b takes part in M2 polarization and it is upregulated upon IL-10 stimulation, and targets interferon regulatory factor 5 (IRF5). In a mouse model, knock-out of miR-146a led to colitis, which could be ameliorated upon administration of miR-146a mimic [151]. Another example of microRNAs promoting M2 phenotype are miR-181a, miR-223 and miR-125a-5p. Bi et al. showed that overexpression of miR-181a promotes M2 polarization by targeting KLF6 and C/EBPα [164]. IL-4 and IL-13 activates PPARγ, which in turn activates transcription of miR-223—another marker of M2 polarization [197]. Among targets of miR-223 leading to M2 polarization, Pknox1 was identified [171]. M1 macrophages, when transfected with miR-125a-5p, are less potent in exerting bactericidal activity against *Escherichia coli*, which results from targeting Kruppel-like Factor 13 (KLF13) and promotion of M2 polarization [138]. According to Huang et al., the M2 phenotype is favored by IL-16 [147]. IL-16 stimulates macrophage polarization into M2 enhancing IL-10, IL-1a and IL-6 expression, and Mir-145 is involved in this process by targeting IL-16 and stimulating IL-10 synthesis [147].

LPS stimulation, responsible for M1 phenotype differentiation, leads to upregulation of miR-17, miR-20a, and miR-106a, which suppresses SIRPα, resulting in macrophage activation [114]. Expression of let-7c is suppressed by LPS, whereas its upregulation results in a change from the M1 to M2 phenotype by targeting the C/EBP-δ transcription factor [110]. Overexpression of let-7b-5p also promotes polarization into the M2 phenotype [109]. MiR-26a also regulates the CREB-C/EBPβ signaling axis, and its downregulation in *Mycobacterium tuberculosis* (Mtb) infection favors M2 polarization [126]. Interestingly, during Mtb infection, miR-26a is downregulated, leading to upregulation of KLF4 and stimulation of the M2 phenotype [198]. KLF4, a transcription factor that drives M2 polarization and regulates the expression of interleukin-10, is a crucial factor of the polarization course [199] and is targeted by miR-26a. Expression of miR-33 is also overexpressed in M1, thus promoting pro-inflammatory functions [132].

The pro-inflammatory phenotype is also induced by prostaglandin E2 (PGE2) which inhibits miR-21a, responsible for regulation STAT3, thus preventing the switch from M1 to M2 macrophages [118]. Huleihel et al. pointed out miR-125b-5p, miR-143-3p, and miR-145-5p as other factors promoting the pro-inflammatory phenotype. Based on total RNA sequencing, they proved activation of a number of proteins and genes responsible for pro-inflammatory cytokine synthesis, and reactive oxygen species production [140]. The overexpression of miR-340 promoted macrophages to have the M1-like phenotype. The authors noticed that miR-340 directly regulates and inversely correlates with CD47, the protein involved in cell migration, adhesion, and apoptosis [174]. A study performed by Tan et al. indicated that CD47/miR-708 regulates tumor-associated macrophage-mediated phagocytosis [180]. On the other hand, Chen et al. correlated CD47-SIRPα pathway activation with the expression level of miR-378a. The authors indicated that miR-378a affects phagocytosis and macrophage differentiation by targeting CD47-SIRPα [175].

The anti-inflammatory phenotype is favored by other microRNAs, such as miR-24. MiR-24 is an example of a negative regulator of macrophage classical activation induced by LPS. The identified mechanism of action involves modulation of phosphoinositide 3-kinase (PI3K) and further inhibition of cytokine synthesis [124]. Qian et al. documented another microRNA, miR-1246, which induces M2 macrophage polarization by targeting TERF2IP via the STAT3 and NF-κB pathways [182]. Similar mechanisms were identified for miR-125a-5p, miR-762, and miR-484, also classified as promoting M2 phenotype [139].

The most recent knowledge highlights the brokerage between polarization and exosomes. Macrophages are capable of secreting miR-21-containing exosomes, thus spreading the reaction to other macrophages [117], mainly through polarization towards the pro-inflammatory phenotype [128] as in the case of miR-27a. MicroRNAs secreted in exosomes by stromal cells can affect macrophages. For instance, mesenchymal stromal cells exposed to *Pseudomonas aeruginosa* secrete miR-466-rich exosomes, which divert macrophages towards M2 polarization via binding with TIRAP [176].

### 4.3. Recognition of Pathogen-Associated Molecular Patterns (PAMPs) by Pattern-Recognition Receptors (PRRs) Expressed on/in Macrophages

PAMPs are highly conserved molecular structures common to pathogenic bacteria, and include lipids, proteins, and nucleic acids, such as lipopolysaccharides (LPS), lipoteichoic acid (LTA), and bacterial DNA. PAMPs are essential for pathogen survival, and usually have unique molecular or subcellular characteristics that are not found in host cells. Therefore, innate immune cells can recognize PAMPs via PRRs and respond to pathogens and their products. PRRs can recognize such molecules, activate natural immunity, and initiate the inflammatory response [200]. PRRS can be classified into five following types based on protein domain homology: Toll-like receptors (TLRs), nucleotide oligomerization domain (NOD)-like receptors (NLRs), retinoic acid-inducible gene-I (RIG-I)-like receptors (RLRs), C-type lectin receptors (CLRs), and are absent in melanoma-2 (AIM2)-like receptors (ALRs). PRRs are basically composed of ligand recognition domains, intermediate domains, and effector domains. PRRs recognize and bind their respective ligands and recruit adaptor molecules with the same structure through their effector domains, initiating downstream signaling pathways to exert effects. Among the mentioned PRRs, TLRs play crucial role in the inflammatory responses to pathogenic infection and have been a target for many studies exploring phagocytosis course [201,202,203].

Some microRNAs have been identified as modulators of TLRs activity e.g., miR-19, -27a, -96, -143, -146a, -155, -185, -203, -223, -590 [204]. They modulate expression of TLR2 and TLR4, as well as scavenger receptors (SR-A, SR-BI, CD36). Scavenger receptors (SRs) are a ‘superfamily’ of membrane-bound receptors that were initially thought to bind and internalize modified low-density lipoprotein (LDL), thus taking part in pathogenesis of atherosclerosis, though it is currently known to bind to a variety of ligands including pathogens. In turn, the influence of miR-181c on TLR4 was confirmed by Zhang et al. in microglia [205].

Some microRNAs were identified as being involved in TLR signaling pathway modulation. Tserel et al. identified miR-1, miR-99b, miR-139-5p, miR-212, miR-218, and miR-511 as overexpressed in macrophages and involved in the toll-like receptor signaling pathway affecting the JAK-STAT cascade, IL-2 production (miR-511), and β-Catenin binding (miR-99b). They also confirmed binding of miR-511 with 3′UTR of TLR4, however, resulting in upregulation of TLR4 in dendritic cells [135].

Stimulation of different TLRs downregulates miR-92a, which targets mitogen-activated protein kinase 4 (MKK4). Consequently, silencing of miR-92a increases the activation of the JNK/c-Jun pathway and stimulates the production of pro-inflammatory cytokines in macrophages [134]. Another example of microRNA upregulated in response to LPS (i.e., via TLR4) is miR-155 [55]. Subsequently, miR-155 targets Src homology-2 domain-containing inositol 5-phosphatase 1 (SHIP1), leading to activation of kinase Akt during the cellular response to LPS [158]. Yao et al. showed that *Streptococcus pneumoniae* endopeptidase O, a virulence protein, upregulates miR-155 expression enhancing TLR2-mediating phagocytosis [159].

LPS stimulation of murine RAW264.7 macrophages resulted in upregulation of miR-155 and down-regulation of miR-125b. Subsequently, miR-155 downregulates IKKε, FADD, and Ripk1, while downregulation of miR-125b derepresses TNF-α (interestingly, upregulation of miR-155 may also lead to upregulation of TNF-α) [141]. On the other hand, upon Mtb infection, contrary changes occur, i.e., up-regulation of miR-125b and down-regulation of miR-155 decreases TNF production [142].

LPS stimulation is negatively modulated by the phosphatidylinositol 3-kinase (PI3K)-Akt pathway, which creates a negative feedback loop important for modulation of the inflammatory response [206]. Androulidaki et al. demonstrated that microRNAs play a role in this phenomenon. Protein kinase Akt1 upregulates let-7e and miR-181c but downregulates miR-155 and miR-125b. They showed that let-7e targets TLR4 and miR-155 targets SOCS1, thus leading to LPS tolerance [57].

Stimulation of TLR2 and TLR4 leads to upregulation of miR-125a-5p in an MYD88-dependent manner and subsequently promotes the M2 phenotype [138], thus taking part in fine-tuning of inflammation.

Viral infection, through TLR signaling and downstream IFN-I receptor-JAK1-STAT1 signal cascade, leads to downregulation of miR-145, which increases production of anti-inflammatory IL-10 [207].

In murine macrophage stimulation of multiple TLRs, namely TLR2, TLR3, and TLR4 by PAM3CSK4, poly(I:C), and LPS, respectively, upregulates miR-147 through NF-κB (+/− IRF3), which suppresses proinflammatory cytokines, such as TNF-α and IL-6 [208].

*Mycobacterium bovis* BCG, a strain used in vaccines, stimulates the TLR2-PI3K-PKCδ-MAPK pathway with subsequent overexpression of miR-155, further resulting in indirect (i.e., miR-155 targets PKI-α, a negative regulator of PKA), upregulation of PUMA, NOXA, BID, BIM, BAK1, and SMAC, and eventually to apoptosis, which results in acquiring immunity [209].

It was shown that in a model of viral infection, miR-155 expression is induced in a TLR/MyD88-independent but retinoic acid-inducible gene I/JNK/NF-κB-dependent pathway, which leads to downregulation of suppressor of cytokine signaling 1 (SOCS1), and thus enhancement of type I IFN signaling, which may exacerbate inflammation [210].

### 4.4. Phagocytosis—Uptake

The uptake course of bacteria has been analyzed using both mouse and human phagocytic cells.

Experimental evaluation confirmed that overexpression of miR-1 decreases phagocytic uptake of *E. coli* by targeting clathrin heavy chain 1 (CLTC1) [111]. Similar observations were noticed for miR-24, miR-30b and miR-142-3p. Overexpression of these microRNAs impaired the uptake of IgG-coated latex beads [123].

Overexpression of miR-146a enhances *E. coli* uptake by THP-1 cells [154], whereas its silencing was demonstrated to decrease *L. donovani* uptake [153]. Upregulation of miR-615-3p increases phagocytosis of *E. coli* by macrophages, while its silencing reduces this. This effect depends on binding with LCoR, which derepresses peroxisome proliferator-activated receptor gamma (PPARγ) [179]

Both bacterial infection and LPS itself induce miR-15a/16. In mice miR-15a/16 knock-out derepresses PU.1, which upregulates TLR4, further modulating Rho GTPase Cdc 42 and TRAF6. These pro-inflammatory effects stimulate both *E. coli* uptake and generation of mitochondrial ROS [113].

In turn, infection with *Listeria monocytogenes*, a pathogen capable of survival within macrophages, results in upregulation of miR-21 in these cells. Knock-out of miR-21 results in an increased bacterial burden, and this effect may be reversed by synthetic miR-21. As in both settings NO production remains unchanged, the difference cannot be explained by impaired pathogen killing. Similarly, miR-21-deficiency stimulates uptake of dextran and *E. coli* bioparticles. MiR-21 takes part in suppressing uptake by downregulating myristoylated alanine-rich C-kinase substrate (MARCKS) and Ras homolog gene family, member B (RhoB); however, whether this regulation is by direct targeting, remains unclear [119].

Zhu et al. reported that LPS induces expression of miR-17, miR-20a, and miR-106a, which collectively target signal regulatory protein alpha (SIRPα), a factor shown previously to inhibit phagocytosis [211]. Indeed, inhibition of the abovementioned microRNAs leads to derepression of SIRPα, resulting in decreased phagocytic capacity measured as zymosan uptake [114]. In experimental conditions zymosan is used to induce experimental inflammation. In macrophages, zymosan-induced responses include the induction of pro-inflammatory cytokines synthesis, arachidonate mobilization, protein phosphorylation, and inositol phosphate formation. Thus, stimulation of the pro-inflammatory response may strengthen the phagocytic ability of macrophages.

The uptake of a pathogen is a dynamic process involving rearrangement of the cytoskeleton. miR-142–3p targets N-wasp, an actin-binding protein regulating actin dynamics during bacterial uptake [145,198]. A negative correlation of miR-142–3p activity and Mtb internalization was caused by targeting PKC alpha, a key regulator involved in phagocytosis [198]. Naqvi et al. confirmed that miR-142-3p directly regulates protein kinase Cα (PKCα), a key gene involved in phagocytosis. Interestingly, miR-142-3p and PKCα exhibit antagonistic expression during monocyte differentiation. The authors also demonstrated that miR-24, miR-30b, and miR-142-3p regulate cytokine production associated with phagocytosis stimulation [125].

Valverde et al. identified miR-142-3p as microRNA targeting three genes (Vinculin, Dab2 and Skap2) directly associated with cytoskeletal rearrangement and cell movement [129]. Another gene involved in rearrangement of cytoskeleton is the ARP2 gene. Padilla et al. showed that mir-124-5p may regulate phagocytosis by targeting the actin cytoskeleton via the ARP2/3 complex [137].

### 4.5. Modulation of Phagosomal Maturation

Mtb induces expression of miR-155, which, by targeting Ras homologue enriched in brain (Rheb), boosts the autophagic response in macrophages, thus promoting the maturation of phagosomes and decreasing the survival rate of intracellular mycobacteria, while transfection with miR-155 inhibitor increases mycobacterial survival. Uptake remains unchanged by miR-155 modulation [162]. Expression of miR-155 is also enhanced in *P. aeruginosa* keratitis, but in this setting the resulting suppression of Rheb decreases both phagocytosis and phagosomal killing of *P. aeruginosa* [163].

Maturation of phagosomes was also stimulated after *Burkholderia pseudomallei* infection and further miR-30b/30c overexpression. The accelerated maturation was caused by targeting Rab32 [130].

### 4.6. Modulation of Reactive Oxygen Species (ROS) Production inside of Phagosomes and Phagolysosomes

Downregulation of miR-23a-3p in macrophages obtained from patients with active pulmonary tuberculosis with high bacterial burden, via targeting IRF1/SP1, leads to inhibition of ROS generation and limitation of phagocytosis efficiency. Both are dependent on the TLR4/TNF-α/TGF-β1/IL-10 signaling pathway, and are suppressed by miR-23a-3p downregulation [122].

Other examples of microRNAs involved in ROS production are miR-30e-5p and miR-302d-3p. Downregulation of miR-30e-5p and miR-302d-3p increased nitric oxide synthase 2 (*Nos2*) mRNA expression and nitric oxide (NO) production [131]. This is strongly influenced by its concentration [212], but in phagocytosis context it acts as a strong antimicrobial and anti-parasite agent.

There are known examples of microRNAs that are overexpressed in macrophages and affect ROS generation. For instance, miR-328 is a key element of the host response to *Haemophilus influenzae* infection as it augments phagocytosis and production of ROS [173]. In turn, increased expression of miR-144 on a rat macrophage model impaired bacterial phagocytic capacity and H_2_O_2_ scavenging ability [146]. ROS production was also enhanced via overexpression by miR-155 [156], which was identified as a potent promoter of M1 polarization. 

It is known that the level of ROS generation is related to the concentration of secreted proinflammatory cytokine; thus, it is not surprising that reduced secretion of the pro-inflammatory cytokines TNF-α and IL-8 and down-regulation of PKC-α suppressed superoxide generation. Both effects are consequences of overexpression of miR-24, miR-30b and miR-142-3p in macrophages [123].

### 4.7. Modulation of Lysosomal Activity

Mtb infection leads to downregulation of miR-26a and subsequent derepression of KLF4, which further impairs trafficking of Mtb to lysosomes, thus contributing to Mtb survival [126]. miR-106-5p, a microRNA upregulated by Mtb infection in macrophages and targets cathepsin S (CtsS) mRNA, thus decreasing lysosomal activity [136,198].

### 4.8. Antigen Presenting

Chaudhuri et al. demonstrated that miR-125b represses IFN regulatory factor 4 (IRF4) and induces CD80 expression, which enhances macrophage antigen presenting cells’ capacities [143].

FcγR-mediated phagocytosis plays an important role in defense against pathogens by the processes of antigen recognition and phagocytosis in macrophages. Luo et al. showed that miR-543 suppresses Fc-gamma receptor (FcγRs) expression limiting contribution of FcγRs to antigen-presentation [213]. Li et al., using porcine alveolar macrophages, performed wide-spread RNA sequencing and proposed a few microRNAs associated with antigen presentation. Five aberrantly expressed microRNAs were found to target phagocytosis-related genes. miR-127 was predicted to regulate SYK kinase, miR-421-3p was predicted to regulate Rac2, and miR-143-3p, miR-199a-5p and miR-1285 were predicted to regulate Vav3. SYK kinase is crucial for FcγR-mediated phagocytosis and is necessary for PI3-kinase activation, and further regulation of Rac2 and Vav3 activity. Rac2 regulates a diverse set of cellular processes including cell growth control, cytoskeleton reorganization, and activation of protein kinases. In turn, Vav3 is one of the major factors regulating cytoskeleton rearrangements [214].

Interestingly, FcγR-mediated phagocytosis may also be modulated by miR-29a/b/c. Insulin like growth factor 1 (Igf1) was identified as the target gene for miR-29a/b/c, and as He et al. suggested, besides modulation of phagocytosis, miR-29a/b/c may also play an important role in progression of brain ischemia [215].

### 4.9. Resolving Inflammation: Conversion to Anti-Inflammatory Phenotype to Terminate Anti-Infectious Response and to Promote Tissue Repair

In general, apoptosis is an essential process for neutrophil functional silencing, removal of emigrated neutrophils, and timely resolution of inflammation. Neutrophils receive survival and pro-apoptotic signals from the inflammatory microenvironment and activate intracellular mechanisms responding to these signals. Apoptotic neutrophils are phagocytosed by macrophages [216], but macrophages also can undergo apoptosis, which may have important clinical consequences, such as atherosclerosis [217].

According to the literature, apoptosis can be modulated by microRNAs. Many microRNAs have been identified as inhibitors of apoptosis e.g., miR-582-5p, miR-223 and let-7b-5p. miR-582-5p and miR-223 inhibited apoptosis by suppression FOXO1 and FOXO3. In turn, let-7b-5p has been identified as a microRNA targeting the 3′-UTR of Fas, a protein with a central role in the regulation of apoptosis [198].

It is worth mentioning that uptake of apoptotic cells is an important part of efferocytosis. Overexpression of let-7c, by promoting the M2 phenotype, increases phagocytosis of apoptotic cells, whereas its knockdown decreases it [110]. At an injury site, efficient clearance of apoptotic cells by wound macrophages or efferocytosis is a prerequisite for the timely resolution of inflammation. Emerging evidence indicates that miR-21 may regulate the inflammatory response. McCubbrey et al. indicated miR-34a as an important modulator of efferocytosis. MicroRNA-34a negatively regulates efferocytosis via SIRT1 [133]. Babu et al. showed the next microRNA able to affect efferocytosis, miR-126 overexpression, attenuates high glucose-induced impairment of efferocytosis [218]. The increase of efferocytosis consequently leads to resolution of inflammation.

Resolving the inflammatory response is as important as its initiation. It is important to mention that a crucial role is played by activation of specific kinases and transcriptional factors (e.g., NF-κB), and further production of anti-inflammatory cytokines.

Treatment of human peripheral blood mononuclear cells with LPS by MyD88 and NF-κB upregulates miR-21, which downregulates TLR4 via PDCD4 [120]. During wound healing, induction of miR-21 leads to suppression of PTEN and PDCD4, and subsequent inhibition of LPS-induced NF-κB activation and decreased TNF-α expression, thus being anti-inflammatory [121].

It was shown that NF-κB, upon its activation in response to LPS, initiates transcription of miR-9-1, which subsequently downregulates NF-κB [112].

MIR-146 seems to be one of the major negative regulators of the immune response. Mice with miR-146a knock-out develop LPS hypersensitivity, autoimmune disorders, myeloproliferative disorders, and lymphomas [219] due to NF-κB dysregulation [220]. Interestingly, overexpression of miR-146a is crucial for inducing LPS tolerance by suppression of TNF-α [221].

Expression of miR-146a-5p and miR-146b-5p are induced by NF-κB, and they act as negative regulators of TNF receptor-associated factor 6 (TRAF6) and IL-1 receptor-associated kinase 1 (IRAK1) [58], and IRAK2, thus inhibiting retinoic acid-inducible gene I (RIG-I)-dependent type I IFN production [222].

It should be noted that the role of microRNAs can differ between macrophages present in different organs. Lochhead et al. reported that *Borrelia burgdorferi* infection leads to upregulation of miR-146a in murine joint tissue (suppressing IRAK1 and TRAF6, as mentioned before). Lack of miR-146a results in overactivation of NF-κB, increase in myeloid cell recruitment and more severe Lyme arthritis, whereas it does not affect Lyme carditis. Importantly, expression of miR-146a does not affect the number of bacteria in the tissue, whereas macrophage uptake of *B. burgdorferi* is higher in the case of miR-146a knock-out. Overall, the lack of miR-146a impairs resolution of inflammation and leads to joint damage [150]. 

MiR-187 was shown to take part in IL-10-mediated suppression of TNF-α, IL-6, and the p40 subunit of IL-12 release upon TLR4 activation by LPS [223].

MiR-466l is upregulated in the peripheral blood of patients with sepsis, and its levels correlate with risk of death. In a murine model, an interesting role of miR-466l was demonstrated. During the inflammatory response, miR-466l is first overexpressed in neutrophils, acting proinflammatory, and then it is overexpressed in macrophages, leading to increase in prostanoids and specialized proresolving mediators (e.g., resolvin D1 [RvD1] and RvD5). Those mediators resolve inflammation and suppress miR-466l [224]. Interestingly, administration of exosomes containing miR-466 can reduce mortality in *P. aeruginosa* pneumonia through their immunomodulatory function [176].

## 5. Microglia

Microglia take part in brain development, maintenance of neuronal networks, and injury repair, by phagocyting microbes, dead cells, redundant synapses, protein aggregates, and other particulate and soluble antigens. As microglia secrete cytokines, they are important players in neuroinflammatory response. As in case of macrophages, microglia activation may be described as M1, initiated by TLR and IFN-γ signaling pathways, characterized by production of proinflammatory cytokines and chemokines, such as TNF-α, IL-6, IL-1β, IL-12, and CCL2, or M2, initiated by IL-4, IL-13, IL-10, and resulting in production of IL-10, TGF-β, and growth factors [225]. Nevertheless, the distinctiveness of microglia from other immune cells is marked by expression of miR-125b-5p, miR-342-3p, and miR-99a [226].

Freilich et al. used LPS and IL-4 to differentiate a primary culture of murine microglia toward the M1 or M2a phenotype. LPS stimulation resulted in upregulation of miR-155, -297b-5p, -302c, -191, -10b, -105, -495, -7a, -670, -1934, -201, -200c-5p, -214-5p, -673, and -141-5p, whereas expression of miR-1928, -3474, -383, -192, -1939, -466b-3p, -2134, -1901, -762, -689, -128-5p, -542, -700, -219, and 705 was lowered. Upon IL-4 stimulation, the authors observed upregulation of miR-145, -297b-5p, and miR-214, accompanied by reduced expression of miR-1939, -711, -1224, -200a-5p, -762, -2138, -2861, -1971, -133a, -2132, -2135, -2133, -124, -2137, and -325 [227].

So far, twenty-five microRNAs have been identified as involved in microglia polarization and modulation of inflammatory functions. They are listed in Table 2 and discussed below. The most widely studied is miR-155. We found five significant papers describing a wide spectrum of its functions.

It was demonstrated that, typical for M1 polarization, upregulation of miR-155 and miR-146a, along with downregulation of miR-124 upon LPS stimulation, is accompanied with increased phagocytic activity. Interestingly, this inflamma-miR profile is also present in microglia-derived exosomes, thus likely contributing to inflammation [242]. Both miR-146a and miR-155 are upregulated in *E. coli*-infected astrocytes, taking part in fine-tuning of inflammatory response by targeting IRAK1 and TRAF6 (miR-146a), and TAB2 (miR-155), and collectively inhibiting the EGFR–NF-κB signaling pathway. Experimental suppression of these microRNAs upon *E. coli* infection aggravated astrocyte and microglia activation and decreased mouse survival time without affecting bacterial loads [236]. Such a negative modulation of innate immune responses by miR-155, with a beneficial antiviral response, was also observed in a Japanese encephalitis virus infection model [243].

Cardoso et al., based on findings in other myeloid-derived cells, demonstrated that miR-155 is induced by LPS in a mouse N9 microglia cell line and in primary cultures. Similar to other authors [244], they confirmed targeting of the suppressor of the cytokine signaling 1 gene (SOCS1) by miR-155. They demonstrated that experimental inhibition of miR-155 results from IFN-β, TNF-α, IL-6 and NO production, as well as CD11b expression, upon LPS stimulation, and that inhibition of miR-155 prevents neuronal death following microglia activation [237].

It was further demonstrated that in vivo inhibition of miR-155 in mice during an experiment on strokes leads to derepression of SOCS-1, SHIP-1 (interaction demonstrated in [158]) and C/EBP-β (interaction demonstrated in [245]) and increased phosphorylation levels of cytokine signaling regulator STAT-3. These changes result in upregulation of anti-inflammatory cytokines, namely IL-10, IL-4, IL-6, MIP-1α, IL-5, and IL-17 [246]. Another report confirmed improved functional recovery through promotion of improved blood flow and microvascular integrity, and reduction of infarct size measured in magnetic resonance imaging [247]. Hypoxia and glucose deprivation leads to downregulation of miR-181c, which leads to derepression of TLR4 and activation of NF-κB signaling [205]. Consequently, forced expression of miR-181c may also have a protective role in stroke, but further studies are needed to test this hypothesis.

p53 is a well-known cell cycle regulator, but it also acts as an effector of immune response in microglia. Su et al. demonstrated that stimulation with IFNγ, IL-1α and MARCO, but not with IL-1β, leads to activation of p53, and subsequent upregulation of miR-155, which in turn downregulates c-Maf, thus inducing an inflammatory response. Simultaneously, p53 upregulates miR-34a and miR-145, both of which regulate Twist2, an activator of c-Maf. The significance of the former pathway was confirmed using middle cerebral artery occlusion, a model of CNS ischemia [230].

MiR-155 may impair β-amyloid1-42 clearance, as its knock-out enhances transmembrane transport of fibrillar β-amyloid and promotes directing it towards low-pH compartments for subsequent catabolism [239].

MiR-155 is upregulated in M1-polarized macrophages and microglia, and in those cells in patients with multiple sclerosis [248]. It is also upregulated in astrocytes from active multiple sclerosis lesions (along with miR-34a and miR-326), contributing to reduced expression of CD47. As CD47 physiologically inhibits phagocytosis, dysregulation of those microRNAs may affect the course of the disease [249]. Indeed, it was shown that knock-out of miR-155 ameliorates experimental autoimmune encephalomyelitis in a murine model of multiple sclerosis [250].

Increased expression of miR-155, with concomitant downregulation of numerous genes, namely P2ry12, Tmem119, Olfml3, Egr1, Atf3, Jun, Fos, Mafb, Csf1r, Tgfb1 and Tgfbr1, which resulted in suppressed phagocytosis, was also found in SOD1 mice, a murine model of amyotrophic lateral sclerosis. Those changes may be reversed by miR-155 ablation, which results in disease amelioration. As upregulation of miR-155 was also identified in human sporadic and familial amyotrophic lateral sclerosis, the authors postulate the possible clinical utility of silencing miR-155 [238].

It was shown that neuronal damage, as in a murine model of traumatic brain injury, leads to overexpression of miR-21-5p [251]. A similar effect was observed in PC12, a rat neuronal cell line, after scratch injury. Overexpressed miR-21-5p was delivered using exosomes to BV2 microglia cells and promoted M1 polarization (mirrored by iNOS activity). Subsequent release of pro-inflammatory factors inhibited neurite outgrowth, increased accumulation of P-tau and promoted the apoptosis of PC12 cells, thus aggravating the damage [228].

A team led by Lukiw showed that miR-34a targets a triggering receptor expressed in myeloid/microglial cells-2 (TREM2). Using retina samples, they confirmed that expression of miR-34a was higher in those obtained from patients with age-related macular degeneration (AMD), whereas TREM2 expression was lower. Using C8B4 mice microglia cells, the authors demonstrated that overexpression of miR-34a impaired phagocytosis of Aβ42 peptides, derivatives of beta-amyloid precursor protein (βAPP), thus contributing to AMD progression [229].

In a murine model of ischemic stroke, it was demonstrated that surviving neurons synthesize miR-98, which is loaded into extracellular vesicles and transferred to microglia. Overall, this prevents stressed but-viable neurons from microglial phagocytosis, at least partially by targeting platelet activating factor receptor in microglia [231]. Similarly, other studies showed that miR-98 takes part in reducing ischemia/reperfusion damage due to its positive influence on the tightness of the blood-brain barrier and reduction of the prevalence of proinflammatory Ly6CHI leukocytes and M1 microglia within the impacted area [252]. Additionally, miR-98 reduces the endothelial pro-inflammatory response by direct targeting monocyte chemotactic protein-1 (MCP-1)/CCL2 and CCL5/RANTES [253].

MiR-124 was identified in microglia, but not in other macrophages. In a zebrafish model it was demonstrated that overexpression of miR-124 reduces microglia motility and phagocytosis, which resulted in accumulation of residual apoptotic cell bodies in the optic tectum [234]. In murine microglia, miR-124 downregulates C/EBP-α (and subsequently PU.1, its downstream effector), and thus induces microglia quiescence in the central nervous system. In experimental autoimmune encephalomyelitis, a murine model of multiple sclerosis, symptoms may be completely prevented by miR-124 overexpression [232]. Caldeira et al. showed in a murine model that microglia aging leads to decreased activation of NF-κB, which impairs phagocytic activity, and these changes are accompanied by decreasing expression of miR-124 and miR-155 [254]. 

Talebi et al. found that expression of both products of mir-142 precursor, namely miR-142-5p and miR-142-3p, are increased in the central nervous system of both patients with multiple sclerosis and animals with experimental autoimmune encephalomyelitis. The authors postulate their capacity of modulating the immune response by targeting SOCS1 and TGFBR1, respectively. Additionally, they demonstrated that transfection of splenocytes with miR-142-5p mimics the promoted differentiation toward the Th1 subtype [235].

MiR-223 is associated with the M2 phenotype. Its knock-out in experimental autoimmune encephalomyelitis mice led to delayed onset of the disease, but without affecting its severity. However, absence of the miR-223 impairs M2 polarization and phagocytosis, which results in vivo in reduced myelin debris clearance [240].

As discussed above, the direction of phenotype polarization is mainly cytokine-dependent. Yip et al. showed that docosahexaenoic acid (DHA) also affects the phenotype by stimulation of the M2 phenotype. The observed mechanisms resulted from upregulation of miR-124 [233]. It was shown that DHA may lessen spinal cord injury [255].

Dexmedetomidine is an α2-adrenoceptor agonist that exerts sedative, analgesic, and opioid-sparing effects, and is used for short- and longer-term sedation in an intensive care setting [256]. Using a BV2 cell line, it was demonstrated that its anti-inflammatory effect is mediated by miR-340, which targets NF-κB, and thus suppresses pro-inflammatory cytokines (TNF-α, IL-6, IL-1β, IL-2 and IL-12), inducing anti-inflammatory IL-10, and decreasing phagocytosis [241]. 

## 6. Osteoclasts

As osteoclasts develop from fused macrophages, osteoclasts are considered to be a component of the mononuclear phagocyte system [257,258]. Formation of osteoclasts from macrophages is principally regulated by macrophage colony-stimulating factor, RANK ligand (RANKL), and osteoprotegerin (OPG) [259]. Interestingly, in mice, miR-21 knock-out results in a decreased number and resorption activity of osteoclasts, causing decreased bone-loss that is both age and estrogen-deficiency-related (Table 3). As such, downregulation of miR-21 may be beneficial in osteoporosis [260]. The effects seem to result from interaction of miR-21 with programmed cell death 4 (PDCD4) [261].

## 7. Neutrophils

Neutrophils are among the key elements of the innate immune system. These complex cells are capable of a significant array of specialized functions and are able to regulate many processes such as acute injury and repair, cancer, autoimmunity, and chronic inflammatory processes. They also aid the development of specific adaptive immune responses and guide the subsequent adaptive immune response [262]. We identified only three original studies that tested the influence of selected microRNAs on phagocytic function of neutrophils, and list them in Table 4. None of the studies identified exact targets of the microRNAs tested. Two of these studies employed transgenic mice, and seem to be in line with our experience that short half-life, inability to cryopreserve or expand them in vitro, and vulnerability to transfectants may be important obstacles in performing further research.

## 8. Retinal Pigment Epithelium

The retinal pigment epithelium (RPE) consists of cells that, among others, perform phagocytosis of photoreceptor outer segment membranes, which make them necessary for proper vision. The influence of microRNAs on retinal pigment epithelial functions was discussed recently [264]. Below, and in Table 5, we focus on particles affecting phagocytosis.

Choi et al., based on in silico studies, showed that inhibition of miR-410 stimulates differentiation of RPE-like cells that perform efficient phagocytosis from umbilical cord blood-derived mesenchymal stem cells [274].

A few studies have focused on microRNAs deregulated in RPE obtained from patients suffering from age-related macular degeneration (AMD). Expression of miR-184 is lower in RPE from AMD patients than healthy donors. This lower level leads to derepression of its target, ezrin, which, in turn, downregulates lysosomal-associated membrane protein 1, impairing phagocytosis and contributing to vision loss [266]. Lack of miR-211 also results in upregulation of ezrin and subsequent defective lysosomal biogenesis and degradative capacity. On the other hand, its upregulation results in increased lysosome number and stimulates their fusion with phagosomes [270]. In mice, knock-out of miR-204 leads to derepression of Rab22a, which physiologically suppresses endosomal maturation. However, the final effect is a blockage of phagolysosomal activity; again, downregulation of miR-204 is observed in AMD [268]. MiR-302d-3p is overexpressed in RPEs of AMD patients. It targets products of CDNK1A and p21Waf1/Cip1, inducing RPE dedifferentiation, cell cycle progression, proliferation, migration, and inhibiting phagocytosis [272]. Similarly, upregulation of miR-382-5p may contribute to pathogenesis of AMD through promotion of dedifferentiation, proliferation, and reactive oxygen species production, whereas phagocytosis is impaired. This microRNA is regulated by circNR3C1, which acts as an endogenous sponge [273]. In a rat model, sodium iodate treatment leads to upregulation of miR-25 (via STAT3), which subsequently downregulates IGTAV and PEDF, and consequently impairs phagocytosis. As such, miR-25 contributes to vision loss in a way similar to that observed in AMD (however, the authors did not report miR-25 level in AMD patients) [265]. Different methodology was used by Tang et al., who used mice with knock-out of Mertk, which is a phagocytosis regulator, to investigate changes in patterns of microRNA expression in RPE. They identified deregulated microRNAs that putatively target genes involved in cytoskeletal regulation; however, functional studies are lacking [275].

MiR-194 is physiologically expressed in rat RPE. It targets zinc finger E-box binding homeobox 1 (ZEB1) and is in silico predicted to regulate numerous cell functions, among others, phagocytosis. Indeed, exogenous administration of miR-194 alleviates proliferative vitreoretinopathy [267].

## 9. Vascular Smooth Muscle Cells

Vascular smooth muscle cells are able to perform nonprofessional phagocytosis and take part in the development of atherosclerotic plaques [106]. It was shown that expression of miR-145, which positively regulates myocardin, is decreased in atherosclerotic lesions (Table 6) [276]. These changes lead to dedifferentiation of vascular smooth muscle cells and their increased phagocytic function [277].

## 10. Conclusions

Phagocytosis is one of the fundamental processes performed by innate immune cells. The advanced machinery involved in phagocytosis of pathogens, dead cells and tissue debris is highly coordinated by intra and extracellular signals. The interest in the role of microRNAs in modulation of immune cells functions, including phagocytosis, has seen a recent surge. In this review we focus on microRNAs which play a regulatory role in phagocytosis. Discerning analysis of all available data on the influence of microRNAs on phagocytosis performed by macrophages, microglia, osteoclasts, neutrophils, retinal pigment epithelium, and vascular smooth muscle cells highlights new perspectives of the modulation of this process. To validate the beneficial effect of the s microRNAs, clinical trials are expected. A successful future approach could result in the modulation of microRNA expression effects on phagocytosis and potentially more effective treatment of infectious diseases.

## Figures and Tables

**Table 1:** MicroRNAs involved in regulation of phagocytosis performed by macrophages.

MicroRNA	Organism	Cell	Setting	Target	Effect	Ref.
let-7a-5p	human	monocytes	downregulated in macrophages compared to monocytes	WASL and VASP	enhanced phagocytosis	[107]
let-7b-5p	human	THP-1	*M. tuberculosis* infection	FAS	inhibition of let-7b-5p augments apoptosis and pathogen clearance	[108]
let-7b-5p	human	monocytes	*S. aureus* infection	SOCS1/STAT	regulates M2 polarization	[109]
let-7c	mice	bone marrow-derived macrophages (M1 and M2)	bleomycin-induced pulmonary fibrosis	C/EBP-δ	let-7c promotes M2 polarization and stimulates phagocytosis of apoptotic cells, whereas its knock-out leads to M1 polarization	[110]
let-7e	mice	RAW264.7	LPS stimulation	TLR4	let-7e is upregulated upon LPS stimulation and targets TLR4 to modulate inflammatory response	[57]
let-7i-5p	human	monocytes	downregulated in macrophages compared to monocytes	WASL and VASP	enhanced phagocytosis	[107]
miR-1	mice	RAW264.7	experimental overexpression	clathrin heavy chain 1 (CLTC1)	decrease of E. coli uptake	[111]
miR-9-1	human	blood monocyte	LPS stimulation	NFKB1	negative feedback on pro-inflammatory response	[112]
miR-15a/16	miR-15a/16 knocked-out mice	bone marrow-derived macrophages	exposure to *E. coli*	derepression of PU.1 after miR-15a/16 knock-out	increased *E. coli* uptake and generation of mitochondrial reactive oxygen species in miR-15a/16 knocked-out mice	[113]
miR-17	human	HL-60, U937, THP-1	LPS-induced upregulation of miR-17, miR-20a, and miR-106a	SIRPα	decreased migration, zymosan particles uptake, and secretion of pro-inflammatory cytokines upon simultaneous microRNAs inhibition	[114]
miR-20a-5p	human	HL-60, U937, THP-1	LPS-induced upregulation of miR-17, miR-20a, and miR-106a	SIRPα	decreased migration, zymosan particles uptake, and secretion of pro-inflammatory cytokines upon simultaneous microRNAs inhibition	[114]
miR-20a-5p	human	monocytes, THP-1	*M. tuberculosis* infection	JNK2	expression of miR-20a-5p is reduced upon infection, which enhance pathogen clearance	[115]
miR-20b-5p	mice	RAW264.7	*M. tuberculosis* infection	Mcl-1 (direct interaction not confirmed)	expression of miR-20b-5p is reduced upon infection, which enhance pathogen survival	[116]
miR-21	mice	RAW264.7	miR-21 transfection, induction by miR-21-rich exosomes	not specified	polarization towards M1 phenotype	[117]
miR-21	miR-21 knock-out mice	peritoneal macrophages	miR-21 is downregulated through PGE2	STAT3 (suppressed by miR-21)	promoting M2 over M1 polarization upon miR-21 knock-out	[118]
miR-21	miR-21 knock-out mice	bone marrow-derived macrophages	miR-21-deficient mice exposed to *L. monocytogenes*	myristoylated alanine-rich C-kinase substrate (MARCKS) and Ras homolog gene family, member B (RhoB)—upregulated in miR-21 knock-out mice; lack of experimental confirmation of direct binding of microRNA with 3′UTRs	increased uptake of *L. monocytogenes*, *E. coli* and dextran	[119]
miR-21	human, mice	bone marrow-derived macrophages, RAW264.7, PDCD4 knock-out mice	LPS stimulation	PDCD4	induction of miR-21 protects from LPS-mediated overstimulation	[120]
miR-21	human	THP-1, bone marrow-derived macrophages	wound healing	PTEN, PDCD4	expression of miR-21 upon LPS stimulation is higher in macrophages performing efferocytosis; miR-21 promotes resolving of inflammation through suppression of NF-κB and induction of IL-10	[121]
miR-23a-3p	human	bone marrow-derived macrophages	*M. tuberculosis* infection	IRF1/SP1	reduction of reactive oxygen species generation and inhibition of TLR4/TNF-α/TGF-β1/IL-10 signaling pathway	[122]
miR-24	human	monocyte	*E. coli* infection, IgG-opsonized beads infection	PKC-α	reduced secretion of TNF-α and IL-8, suppressed superoxide generation and reduction in expression of FcRs including FCGR2A, FcɛR1G and FCER2	[123]
miR-24	human	monocyte	LPS stimulation	p110δ	reduced secretion of cytokines, and promotion of anti-inflammatory phenotype	[124]
miR-24	human	monocytes	*E. coli* and *S. aureus* infection	PKCα	modulation of phagocytosis and cytokine production	[125]
miR-26a	human, mice	bone marrow-derived macrophages, RAW264.7	*M. tuberculosis* infection	KLF4	downregulation of miR-26a promotes M2 polarization and intracellular pathogen survival due to decreased trafficking to lysosomes	[126].
miR-26a	rat	bone marrow-derived macrophages	co-culture with dying cells	C1qa	promotion of M1 phenotype	[127]
miR-27a	human	monocytes, THP-1	alcohol-exposed monocytes	not specified	monocytes polarize into M2 macrophages as indicated by increased surface expression of CD68 (macrophage marker), M2 markers (CD206 (mannose receptor) and CD163 (scavenger receptor)), secretion of IL-10, and TGFβ and increased phagocytic activity	[128]
miR-30b	human	monocyte	*E. coli* infection, IgG-opsonized beads infection	PKCα	reduced secretion of TNF-α and IL-8, suppressed superoxide generation and reduction in expression of FcRs including FCGR2A, FcɛR1G and FCER2	[123]
miR-30b	human	monocytes	*E. coli* and *S. aureus* infection	PKCα	modulation of phagocytosis and cytokine production	[125]
miR-30b	human	monocytes	experimental overexpression	Vinculin, Dab2 and Skap2 directly associated with cytoskeletal rearrangement	regulation of cytoskeletal rearrangement and cell movement	[129]
miR-30b/30c	mice	RAW264.7	*B. pseudomallei* infection	Rab32	enhanced phagosome maturation	[130]
miR-30e-5p	mice	BALB/c macrophages	*L. amazonensis* infection	increased nitric oxide synthase 2 (*Nos2*) mRNA expression levels and nitric oxide (NO) production	nitric oxide is secreted as free radicals in an immune response and is toxic to intracellular parasites	[131]
miR-33	mice macrophages’ specific miR-33 inhibition	bone marrow-derived macrophages	inflammation in atherosclerotic plaque	AMP-activated protein kinase and retinoic acid-producing enzyme aldehyde dehydrogenase family 1, subfamily A2 (direct or indirect)	increased oxidative respiration, promoted M2 polarization and reduction of atherosclerotic plaque upon miR-33 knock-out (partially due to Treg lymphocyte-mediated effects)	[132]
miR-34a	mice	C57BL/6	induction of apoptosis by dexamethasone treatment	SIRT1	negatively regulates efferocytosis	[133]
miR-92a	mice	RAW264.7, *MyD88* knock-out mice	stimulation of multiple TLRs (mainly TLR4 by LPS), which leads to downregulation of miR-92a	mitogen-activated protein kinase kinase 4 (MKK4)	TLR-mediated miR-92a derepresses production of pro-inflammatory cytokines and impedes resolution of inflammation	[134]
miR-99b	human	monocytes	experimental differentiation into macrophages or dendritic cells	TLR4	reduced differentiation into dendritic cells	[135]
miR-106a	human	HL-60, U937, THP-1	LPS-induced upregulation of miR-17, miR-20a, and miR-106a	SIRPα	decreased migration, zymosan particles uptake, and secretion of pro-inflammatory cytokines upon simultaneous microRNAs inhibition	[114]
miR-106b-5p	human	monocytes	*M. tuberculosis* infection	cathepsin S (CtsS)	decreased host lysosomal enzymatic activity	[136]
mir-124-5p	human	monocytes	experimental overexpression	ARP2/3 complex	rearrangement of actin cytoskeleton	[137]
miR-125a-5p	mice	bone marrow-derived macrophages, KLF13 knock-out mice	TLR2 and TLR4-dependent upregulation of miR-125a-5p	Kruppel-like Factor 13 (KLF13)	miR-125a-5p upregulation decrease of bactericidal activity, promote switch from M1 to M2 polarization and resolution of inflammation	[138]
miR-125a-5p	mice	bone marrow-derived macrophages, BALB/c mice	stimulation with LPS and *T. crassiceps*-excreted/secreted antigens	not specified	miR-125a-5p upregulation promotes of M2 phenotype	[139]
miR-125b-5p	mice	bone marrow-derived macrophages, C57bl/6 mice	exposition on biomaterials	not specified	miR-125b-5p downregulation promotes of M1 phenotype	[140]
miR-125b-5p	mice	RAW264.7, C57BL/6 mice	LPS stimulation	TNF-α	LPS-induced miR-125b-5p downregulation promotes M1 phenotype via TNF-α production	[141]
miR-125b-5p	human	bone marrow-derived macrophages	*M. tuberculosis* and *M. smegmatis* lipomannan stimulation	TNF-α	Mtb-induced miR-125b-5p upregulation promote M2 phenotype, and repression of TNF-α production, whereas *M. smegmatis* promotes miR-125b-5p downregulation and thus M1 phenotype and enhanced phagocytic capacity	[142]
miR-125b-5p	mice, C57Bl/6 mice	RAW264.7, bone marrow-derived macrophages	experimental overexpression or silencing	IFN regulatory factor 4 (IRF4)	suppression of IRF4 and induction of CD80, what enhances macrophages’ antigen presenting cells capacities	[143]
miR-128	C57BL/6 mice	bone marrow-derived macrophages	co-culturing with Panc02 cells	not specified	increased phagocytosis	[144]
miR-139-5p	human	monocytes	experimental differentiation into macrophages or dendritic cells	TLR4	reduced differentiation into dendritic cells	[135]
hsa-miR-142-3p	human, mice	monocytes, J774A	*M. tuberculosis* infection	N-Wasp, an actin-binding protein involved in actin dynamics during bacterial uptake	modulation of bacteria uptake, decreased internalization	[145]
miR-142-3p	human	monocyte	*E. coli* infection, IgG-opsonized beads infection	PKC-α	reduced secretion of TNF-α and IL-8, suppressed superoxide generation and reduction in expression of FcRs including FCGR2A, FcɛR1G and FCER2	[123]
miR-142-3p	human	monocytes	experimental overexpression	Vinculin, Dab2 and Skap2 directly associated with cytoskeletal rearrangement	regulation of cytoskeletal rearrangement and cell movement	[129]
miR-142-3p	human	monocytes	*E. coli* and *S. aureus* infection	PKCα	modulation of phagocytosis and cytokine production	[125]
miR-143-3p	mice	bone marrow-derived macrophages, C57bl/6 mice	exposition on biomaterials	not specified	promotion of M1 phenotype	[140]
miR-144	rat	macrophages	HIV infection	Nrf2	impaired bacterial phagocytic capacity and H_2_O_2_ scavenging ability	[146]
miR-145-5p	mice	bone marrow-derived macrophages, C57bl/6 mice	exposition on biomaterials	not specified	promotion of M1 phenotype	[140]
miR-145-3p	human	THP-1	LPS stimulation	not specified	promotion of M2 polarization	[147]
miR-146a	mice	RAW264.7	experimental overexpression	TLR2	SNP in miR-146a affects regulation of expression of TLR2, which regulates amyloid uptake, and may contribute to the risk of Alzheimer’s disease	[148]
miR-146a	human	THP-1	macrophages of atherosclerotic plaque	TLR4	overexpression of miR-146a reduces intracellular LDL cholesterol content and secretion of interleukin 6, interleukin 8, chemokine (C-C motif) ligand 2 and matrix metallopeptidase 9, thus may suppress atherosclerosis	[149]
miR-146a	miR-146a knock-out mice	macrophages	*B. burgdorferi* infection	overactivation of NF-κB (due to miR-146a knock-out)	increased pathogen uptake, impaired resolving of inflammation and more severe Lyme arthritis	[150]
miR-146a	IL-10 and Rag1 double knock-out mice	bone marrow-derived macrophages	colitis model	interferon regulatory factor 5 (IRF5)	miR-146a knock-out leads to M1 polarization and intestinal inflammation, whereas treatment with miR-146a mimic ameliorates colitis	[151]
miR-146a	mice	RAW264.7	experimental overexpression or silencing	Notch1, Peroxisome proliferator-activated receptor γ (PPARγ) (directly or indirectly)	M2 polarization	[152]
miR-146a	mice	bone marrow derived macrophages	*L. donovani* infection	TRAF6, IRAK1	M2 polarization, decreased phagocytosis of *L. donovani* upon miR-146a inhibition	[153]
miR-146a	human, mice	THP-1, C57BL/6.NOD-Aec1Aec2 (mouse model of Sjögren’s syndrome)	verification of the role of miR-146a, which is upregulated in Sjögren’s syndrome patients (and mice model)	not specified	increased uptake of *E. coli* and suppression of pro-inflammatory cytokine production upon miR-146a overexpression	[154]
miR-155	human	THP-1	experimental overexpression or silencing	SCG2	overexpression of miR-155 decrease lipid uptake, potentially affecting atherosclerosis	[155]
miR-155	human	monocytes	experimental overexpression or silencing	not specified	increased ROS production, and M1 phenotype promotion	[156]
miR-155	human	monocytes	*Vibrio anguillarum* infection	not specified	M1 phenotype promotion	[157]
miR-155	mice	RAW264.7, C57BL/6 mice	LPS stimulation	Fas-associated death domain protein (FADD), IkappaB kinase epsilon (IKKepsilon), and the receptor (TNFR superfamily)-interacting serine-threonine kinase 1 (Ripk1)	LPS stimulation upregulates miR-155, thus increases TNF-α production	[141]
miR-155	mice	RAW264.7	LPS stimulation	Suppressor of Cytokine Signaling 1 (SOCS1)	miR-155 is downregulated upon LPS stimulation, which derepresses SOCS1 to modulate inflammatory response	[57]
miR-155	mice	RAW264.7	experimental overexpression or silencing	Src homology-2 domain-containing inositol 5-phosphatase 1 (SHIP1)	increased activation of Akt upon LPS stimulation	[158]
miR-155	human	bone marrow-derived macrophages	*M. tuberculosis* lipomannan stimulation	SH-2 containing inositol 5′ polyphosphatase 1 (SHIP1)	lipomannan stimulation downregulates miR-155, thus derepressing SHIP1, consequently downregulating TNF-α	[142]
miR-155	mice	C57 or TLR2KO mice	*S. aureus* or *S. pneumoniae* infection	SH-2 containing inositol 5′ polyphosphatase 1 (SHIP1)	enhanced bacteria uptake	[159]
miR-155	mice	miR-155 knock-out mice	macrophages of atherosclerotic plaque	BCL6	mildly oxidized LDL increases expression of miR-155, which downregulates BCL6, thus attenuating NF-κB signaling	[160]
miR-155	human	monocytes	co-culture with abnormal red blood cell	BACH1	enhanced phagocytic activity	[161]
miR-155	mice	RAW264.7, bone marrow-derived macrophages	induction of miR-155 by *M. tuberculosis* infection	Ras homologue enriched in brain (Rheb)	promotion of maturation of mycobacterium-containing phagosomes and decreasing the survival rate of intracellular mycobacteria	[162]
miR-155	human, mice	corneas, RAW264.7, bone marrow-derived macrophages	induction of miR-155 in *P. aeruginosa*–induced keratitis	Ras homologue enriched in brain (Rheb)	suppression of phagocytosis and intracellular killing of *P. aeruginosa*	[163]
miR-181a	human, mice	THP-1, RAW264.7	experimental overexpression or silencing	KLF6, C/EBPα	M2 polarization	[164]
miR-181b	human	monocytes	zymosan stimulation	ALX/FPR2	stimulated phagocytic activity	[165]
miR-181b	human	monocytes	zymosan stimulation or *P. aeruginosa* infection	not specified	modulation of receptor-dependent LXA4-induced phagocytosis	[166]
miR-183	human	monocytes	*M. tuberculosis* infection	NF-κB	increased phagocytosis	[167]
miR-183/96/182 cluster	mice	miR-183/96/182 knockout mice, RAW264.7	*P. aeruginosa*-induced keratitis	not specified	knock-out and experimental silencing increases phagocytic capacity, knock-out decreases inflammatory response and severity of keratitis	[168]
miR-185-5p	human, mice	THP-1, RAW264.7	phagocytosis of intrarenal CaOx crystals	not specified	stimulation of M2 phenotype	[169]
miR-200a	human	monocytes	co-culturing with nasopharyngeal carcinoma cells	CD47	increased phagocytosis	[170]
miR-212	human	monocytes	experimental differentiation into macrophages or dendritic cells	TLR4	reduced differentiation into dendritic cells	[135]
miR-218	human	monocytes	experimental differentiation into macrophages or dendritic cells	TLR4	reduced differentiation into dendritic cells	[135]
miR-223	miR-223 knock-out mice	bone marrow derived macrophages	obesity-associated adipose tissue inflammation	Pknox1	preferential M1 polarization and exacerbation of insulin resistance and adipose tissue inflammation in miR-223-deficient mice	[171]
miR-223	human	monocytes	*M tuberculosis* infection	FOXO3	suppressed apoptosis of macrophages	[172]
miR-302d-3p	mice	BALB/c mice macrophages	*Leishmania amazonensis* infection	increased nitric oxide synthase 2 (*Nos2*) mRNA expression levels and nitric oxide (NO) production	nitric oxide is secreted as free radicals in an immune response and is toxic to intracellular parasites	[131]
miR-328	mice, human	monocytes	*H. influenzae* infection	not specified	miR-328 inhibition augments phagocytosis and production of reactive oxygen species	[173]
miR-340	mice	bone marrow derived macrophages of C57BL/6 mice	co-culture macrophages with pancreatic cancer cells	not specified	stimulation of M1 phenotype	[174]
miR-378a	mice	ApoE^−/−^ mice macrophages	zymosan stimulation, or co-culture with Ishikawa cells	SIRPα	modulation of phagocytosis and differentiation	[175]
miR-466	human	monocytes	*P. aeruginosa* infection	TIRAP	miR-466 delivery in extracellular vesicles promotes M2 polarization, increases pathogen phagocytosis, suppresses pro-inflammatory factors, decreases neutrophil efflux, and reduces infected mice mortality	[176]
miR-484	mice	bone marrow-derived macrophages of BALB/c mice	stimulation with LPS and *T. crassiceps*-excreted/secreted antigens	not specified	promotion of M2 phenotype	[139]
miR-511	human	monocytes	experimental differentiation into macrophages or dendritic cells	TLR4	reduced differentiation into dendritic cells	[135]
miR-582-5p	human	monocytes, THP-1	*M. tuberculosis* infection	FOXO1	suppressed apoptosis of macrophages	[177]
miR-590	apoE^−/−^ mice	macrophages	experimental overexpression and silencing	lipoprotein lipase	miR-590 decreases concentration of proinflammatory cytokines and atherosclerotic plaque	[178]
miR-615-3p	human	THP-1, splenic macrophages	hypersplenism (resulting in overexpression of miR-615-3p	ligand-dependent nuclear receptor corepressor (LCoR), which is a derepressor of peroxisome proliferator-activated receptor gamma (PPARγ)	inhibition on miR-615-3p reduces uptake of E. coli	[179]
miR-708-5p	BALB/c mice	macrophages	co-incubation with CFSE-labelled breast cancer cells	CD47	enhanced phagocytosis	[180]
miR-708-5p	human	THP-1, U937	*M. tuberculosis* infection	TLR4	miR-708-5p expression increases upon infection and enhances pathogen survival	[181]
miR-762	BALB/c mice	bone marrow-derived macrophages	stimulation with LPS and *T. crassiceps*-excreted/secreted antigens	not specified	promotion of M2 phenotype	[139]
miR-1246	human	monocytes	co-culture with glioma-derived exosomes (GDEs)	TERF2IP	induced M2 polarization	[182]
miR-4270	human	monocytes	*H. pylori* infection	CD300E	induced M1 polarization	[183]

**Table 2:** MicroRNAs involved in regulation of phagocytosis performed by microglia.

MicroRNA	Organism	Cell	Setting	Target	Effect	Ref.
miR-21-5p	murine/rat cell lines	PC12 (murine neuronal cell line)	experimental overexpression	BV2 (rat microglia cell line)	M1 polarization	[228]
miR-34a	human/mice	C8B4-microglial cells	age-related macular degeneration (AMD)	triggering receptor expressed in myeloid/microglial cells-2 (TREM2)	decreased uptake of Aβ42-peptides	[229]
miR-34a	p53-deficient mice	RAW cell line	experimental overexpression	Twist2	p53-dependent miR-34a upregulation represses Twist2, and consequently anti-inflammatory c-Maf	[230]
miR-98	mice	extracellular vesicles secreted by neurons	murine model of ischemic stroke	platelet activating factor receptor in microglia	prevention of stress-but-viable neurons from microglial phagocytosis	[231]
miR-124	C57BL/6 mice	microglia	experimental autoimmune encephalomyelitis	CCAAT/enhancer-binding protein-α (C/EBP-α)	suppression of inflammation	[232]
miR-124	primary adult rat spinal microglia cultures and in the murine microglial cell line BV2	microglia	spinal cord injury	not specified	reduced myelin phagocytosis	[233]
miR-124	Danio rerio	microglia	experimental silencing and overexpression	not specified	overexpression of miR-124 reduces microglia motility and phagocytosis	[234]
miR-142-5p	human brain autopsy samples, C57BL/6 mice	murine splenocytes	multiple sclerosis, experimental autoimmune encephalomyelitis	SOCS1	promotion of differentiation towards Th1 subtype	[235]
miR-142-3p				TGFBR1		
miR-145	p53-deficient mice	RAW cell line	experimental overexpression	Twist2	p53-dependent miR-34a upregulation represses Twist2, and consequently anti-inflammatory c-Maf	[230]
miR-146	SPF KM mice	U251 human astrocyte cell line	*E. coli* strain PCN033 infection	IRAK1 and TRAF6	fine-tuning of inflammation through negative feedback loop with NF-κB	[236]
miR-155	SPF KM mice	U251 human astrocyte cell line	*E. coli* strain PCN033 infection	TAB2	fine-tuning of inflammation through negative feedback loop with NF-κB	[236]
miR-155	cell line	N9 microglia cells	LPS stimulation	SOCS1	downregulation of inflammatory cytokines and inducible nitric oxide synthase, decreased production of nitric oxide; decreased neurons phagocytosis by activated microglia	[237]
miR-155	p53- or miR-155 deficient mice	microglia	model of neuroinflammation	c-Maf	p53-dependent miR-155 upregulation represses anti-inflammatory c-Maf	[230]
miR-155	SOD1 mice	microglia	miR-155 knock-out	not specified	increased phagocytic function and disease amelioration upon miR-155 knock-out	[238]
miR-155	C57/BL6 wild-type mice	primary microglia	miR-155 knock-out or overexpression	not specified	increased amyloid uptake and catabolism upon miR-155 knock-out	[239]
miR-181c	mice, rat	BV-2, primary microglia	oxygen-glucose deprivation, experimental overexpression and silencing	TLR4	oxygen-glucose deprivation leads to miR-181c downregulation and subsequent derepression of TLR4, promoting inflammatory response	[205]
miR-223	SOD1 mice	macrophages, microglia	miR-155 knock-out	not specified	miR-223 takes part in M2 polarization and stimulates myelin debris clearance	[240]
miR-340	rat cell line	BV2 (microglia cell line)	LPS-induced inflammation attenuated by dexmedetomidine	NF-κB	suppression of pro-inflammatory cytokines (TNF-α, IL-6, IL-1β, IL-2 and IL-12), induction of anti-inflammatory IL-10, and inhibition of phagocytosis	[241]

**Table 3:** MicroRNAs involved in regulation of phagocytosis performed by osteoclasts.

MicroRNA	Organism	Cell	Setting	Target	Effect	Refs.
miR-21	mice	osteoclasts	experimental knock-out	PDCD4	decreased bone loss in miR-21 knock-out mice	[260,261]

**Table 4:** MicroRNAs involved in regulation of phagocytosis performed by neutrophils.

MicroRNA	Organism	Cell	Setting	Target	Effect	Ref.
miR-142-5p and miR-142-3p	mir-142 knock-out mice	neutrophils	*S. aureus* skin wound infection	small GTPases *Rho*, *Rac*, and *Cdc42*	increased bacteria load, impaired abscess formation, and decreased phagocytic activity due to impaired cytoskeleton remodeling in neutrophils of mir-142 knock-out mice	[263]
miR-183/96/182 cluster	miR-183/96/182 knockout mice	neutrophils	*P. aeruginosa*-induced keratitis	not specified	knock-out increases phagocytic capacity, knock-out decreases inflammatory response and severity of keratitis	[168]
miR-328	mice, human	neutrophils	experimental inhibition, *H. influenzae* infection	not specified	miR-328 inhibition augments phagocytosis of *H. influenzae* and increases ROS production	[173]

**Table 5:** MicroRNAs involved in regulation of phagocytosis performed by retinal pigment epithelium.

MicroRNA	Organism	Cell	Setting	Target	Effect	Refs.
miR-25	rat	retinal pigment epithelium	model of age-related macular degeneration	IGTAV and PEDF	decreased phagocytosis, retina degeneration and visual impairment	[265]
miR-184	human	primary retinal pigment epithelium	age-related macular degeneration	ezrin	impaired phagocytosis and visual impairment due to low expression of miR-184	[266]
miR-194	ARPE-19 cell line, rat	primary retinal pigment epithelium	model of proliferative vitreoretinopathy	zinc finger E-box binding homeobox 1 (ZEB1)	miR-194 administration in vivo suppressed proliferative vitreoretinopathy in the rat model	[267]
miR-204	mice, human	retinal pigment epithelium	miR-204 knock-out	Rab22a	impaired phagocytosis and visual impairment due to low expression of miR-204	[268,269]
miR-211	mice, ARPE-19 cell line	retinal pigment epithelium	miR-211 knock-out and overexpression	ezrin	overexpression of miR-211 stimulates lysosomal biogenesis and increase autophagosome–lysosome fusion	[270,271]
miR-302d-3p	HiPSC-RPE, ARPE-19 cell lines	retinal pigment epithelium	miR-302d-3p silencing and overexpression	p21^Waf1/Cip1^	miR-302d-3p induces RPE dedifferentiation, cell cycle progression, proliferation, migration, inhibits phagocytosis	[272]
miR-382-5p	ARPE-19 cell line	retinal pigment epithelium	miR-382-5p silencing and overexpression, direct or mediated via manipulation of circNR3C1	PTEN	miR-382-5p induces RPE dedifferentiation, proliferation, migration, inhibits phagocytosis	[273]

**Table 6:** MicroRNAs involved in regulation of phagocytosis performed by vascular smooth muscle cells.

MicroRNA	Organism	Cell	Setting	Target	Effect	Refs.
miR-145	mice	primary culture of vascular smooth muscle cells	cholesterol loading	myocardin (positive regulation)	increased phagocytic function	[276,277]

## Data Availability

Not applicable.
